# A Prospective Audit Comparing Optos Widefield Imaging to Fundus Examination for Von Hippel-Lindau Retinal Screening

**DOI:** 10.7759/cureus.32814

**Published:** 2022-12-22

**Authors:** Naeem Iqbal, Matthew Stahl, Ernest Lim, Saoud Al-Khuzaei, Rebecca Jones, Dorothy Halliday, Susan Downes

**Affiliations:** 1 Department of Ophthalmology, Queen Alexandra Hospital, Portsmouth, GBR; 2 Department of Ophthalmology, Moorfields Eye Hospital National Health Service (NHS) Foundation Trust, London, GBR; 3 Department of Clinical Neurosciences, University of Oxford, Oxford, GBR; 4 Department of Ophthalmology, Bristol Royal Infirmary, Bristol, GBR; 5 Department of Medicine, Oxford Centre for Genomic Medicine, Nuffield Orthopaedic Hospital, Oxford, GBR; 6 Department of Ophthalmology, John Radcliffe Hospital, Oxford, GBR

**Keywords:** von hippel-lindau disease (vhl), quality improvement, optos ultra-widefield imaging, virtual clinic, retinal haemangioma

## Abstract

Background

Von Hippel-Lindau (VHL) disease is an autosomal dominant multisystem disorder caused by germline mutations at chromosome *3p25-26* in the VHLtumour suppressor gene. Retinal manifestations include capillary haemangiomas that develop in up to 80% of gene carriers. Lifelong retinal surveillance involves yearly assessment usually by fundoscopy and often as part of a VHL multidisciplinary clinic. Optos ultra-widefield retinal imaging is now becoming more widely used in virtual retinal screening clinics. We aimed to assess discrepancies in the pickup rate of angioma and angiomatous-associated disease between slit-lamp fundoscopy and Optos ultra-widefield imaging.

Methodology

A total of 49 patients had both Optos ultra-widefield retinal imaging and slit-lamp fundoscopy over 16 months in VHL retinal surveillance clinics at the John Radcliffe Hospital, Oxford, UK. Optos images were analysed for image quality and presence of angioma(s) by a Consultant Ophthalmologist who was masked to the fundoscopy findings. The pickup rate was compared between slit-lamp fundoscopy and Optos imaging.

Results

In total, data on 94 eyes were collected. Of the total Optos retinal images, 12.8% were positive for angiomas compared to 11.7% from the slit-lamp examination. There was a discrepancy of 1.1% (one value) where the Optos image analysis suggested a possible angioma, which was not identified on slit-lamp examination. Optos imaging identified all angiomas in this cohort.

Conclusions

Optos imaging was non-inferior to slit-lamp examination in this sample of 94 eyes. In the current COVID-19 climate, reducing clinician-patient interaction is important. This research supports providing retinal imaging as an acceptable alternative to the yearly slit-lamp fundus examination.

## Introduction

Von-Hippel Lindau (VHL) disease is a genetic condition with multiple system involvement [1]. Identification of a heterozygous germline VHL pathogenic variant on molecular genetic testing confirms the diagnosis. A pathogenic variant in VHL is highly penetrant, but there is phenotypic variability in expressivity and limited phenotype-genotype correlation. The condition is most commonly associated with retinal and central nervous haemangioblastomas, clear cell renal carcinoma and phaeochromocytomas [2]. Retinal angiomas are the most commonly presenting tumour in VHL disease, with bilateral involvement in 50% of cases [3]. Complications from untreated retinal angiomas include exudation, retinal traction and detachment, and haemorrhage. These complications most commonly occur with larger angiomas. Untreated, these lesions can become sight-threatening and the cumulative risk of visual loss by age 50 years in patients with angiomatosis is 55% [4].

Lesions typically present asymptomatically, and as size increases, management becomes more challenging. Laser or cryotherapy treatment of larger tumours is associated with consequent destruction of the underlying retina, including the surrounding tissue, and may be associated with acute exudative response. This can result in exudative and sometimes tractional retinal detachment and associated visual loss. Regular surveillance to identify new angiomas at the earliest opportunity, with subsequent laser photocoagulation/cryotherapy to ablate the angioma is the current standard management [4]. In the case of angiomas located at the optic disc, longer surveillance may be indicated. Due to the risk of optic nerve damage from treatment, the risks of treatment may outweigh the potential benefit [5].

Standard of care includes multi-system screening appointments with associated imaging regularly to identify disease progression from five years of age and continues lifelong [1]. The retinal examination is carried out to assess for new retinal angiomas and any progression of previously identified angiomas or scarring. Early identification is crucial as prompt and early treatment is associated with lower morbidity. At the Oxford Eye Hospital, individuals with a VHL pathogenic variant or at risk for VHL based on their personal or family history are invited to attend a yearly retinal surveillance programme where the retina is screened for angiomas by a specialist ophthalmologist [6]. Although this study was started before COVID-19, many clinical visits were postponed and face-to-face appointments were reduced; thus, these virtual clinics enabled continued surveillance and crucial early clinical identification of retinal lesions.

We investigated whether Optos ultra-widefield imaging can safely replace the slit-lamp examination, thus enabling a *virtual clinic*. This audit, unlike any previous study, prospectively examined the sensitivities of the slit-lamp fundus examination versus Optos ultra-widefield imaging in a cohort of VHL consecutive patients unstratified for risk. This research was presented as a poster at the Royal College of Ophthalmologists Annual Congress 2021.

## Materials and methods

**Figure 1:**
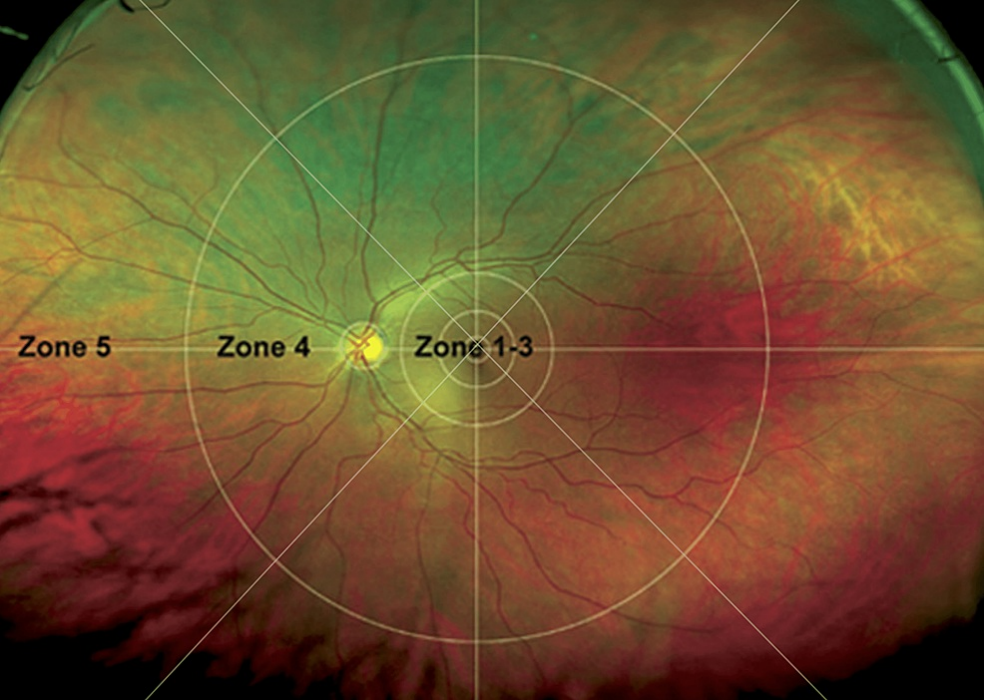
Classification of zones for image quality analysis. Source: [7].

**Figure 2:**
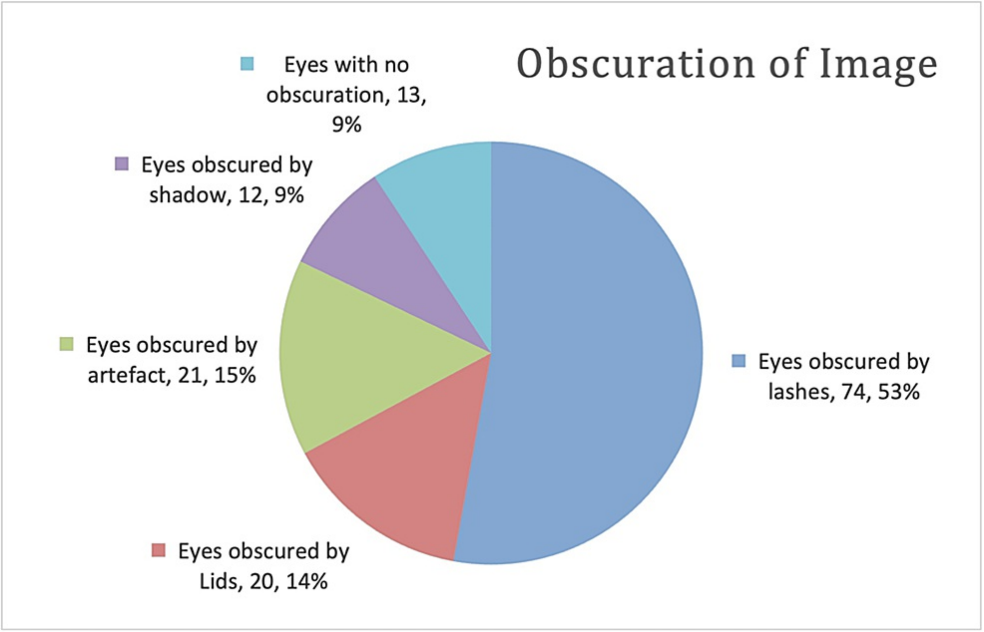
Analysis of the nature of obscuration within Optos ultra-widefield image.

**Figure 3:**
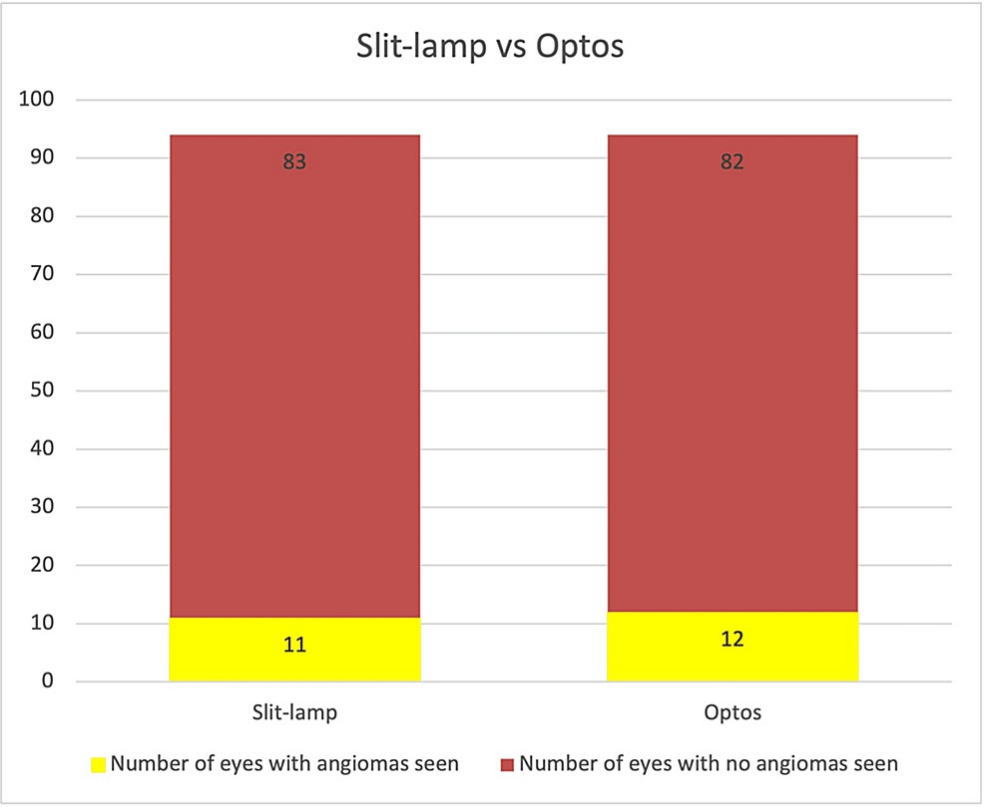
Slit-lamp examination findings versus Optos ultra-widefield image remote analysis.

The Oxford VHL screening service invites VHL patients for yearly retinal disease surveillance. Attendees between October 2018 and February 2020 (12 clinics) were invited for same-day dilated fundus photography using an ultra-widefield Optos camera following the slit-lamp examination by a Medical Retinal Fellow. Patients consented to imaging with an Optos ultra-widefield camera - we used the Daytona Plus 200tx. In images with significant obscuration from lids or lashes, the image was repeated having applied tape to either the upper, lower or both eyelids to increase the camera field of view. A medical photographer was responsible for image capture and was briefed to upload to our system the image that gave the widest field of view. The digital images were remotely graded by a consultant ophthalmologist who was masked to the findings from the slit-lamp examination. Th

e image was first divided into five zones, which were further subdivided into superior, inferior, temporal and nasal regions (Figure 1). The 12 zones were created by dividing zones 1-3 into four regions, zone 4 into another four and zone 5 likewise. These zones were graded for image quality in a binary manner - good quality versus poor quality, whereby poor quality referred to zone obscuration. The cause of obscuration in poor-quality zones was recorded (Figure 2). The image was then assessed for retinal angiomatous disease (Figure 3). The number of angiomatous eyes from the digital images was compared with slit-lamp examination findings to assess for discrepancies.

## Results

A total of 49 patients consented to retinal imaging with the Optos ultra-widefield camera, following the slit-lamp examination, and in total, 94 retinas were imaged (approximately half the patients approached consented to take part in the study, with those not willing to take part largely citing time pressures as the reason). Two patients had prior enucleation, and two others had only one eye imaged owing to communication failure at the time of imaging. Of the total retinal images, 86% had some degree of obstruction; however, 96% of these images had occlusion of 4 or fewer of the 12 retinal zones. The mean number of retinal zones occluded is 2.4/12 (Table 1). Of the 94 eyes, 74 had some degree of occlusion from lashes (79%), 20 eyes had occlusion from lids (21%), 21 eyes had occlusion from artefact (22%) and 12 eyes had occlusion from shadow (13%). Thirteen eyes had no occlusion (14%). This data is demonstrated graphically in Figure 2.

**Table 1:** Analysis of the Optos ultra-widefield image quality. The score represents the proportion of images obscured. The image is divided into 12 segments as per Figure 1. The score represents the number of segments obscured, with a score of 12 representing complete obscuration. No, number; R, right; L, left

Score	No of R eyes	No of L eyes	Total number of eyes
0	5	8	13
1	6	4	10
2	12	14	26
3	13	20	33
4	7	1	8
5	0	1	1
6	1	0	1
7	0	0	0
8	0	0	0
9	0	0	0
10	0	0	0
11	1	0	1
12	1	0	1
Total	46	48	94

**Figure 4:**
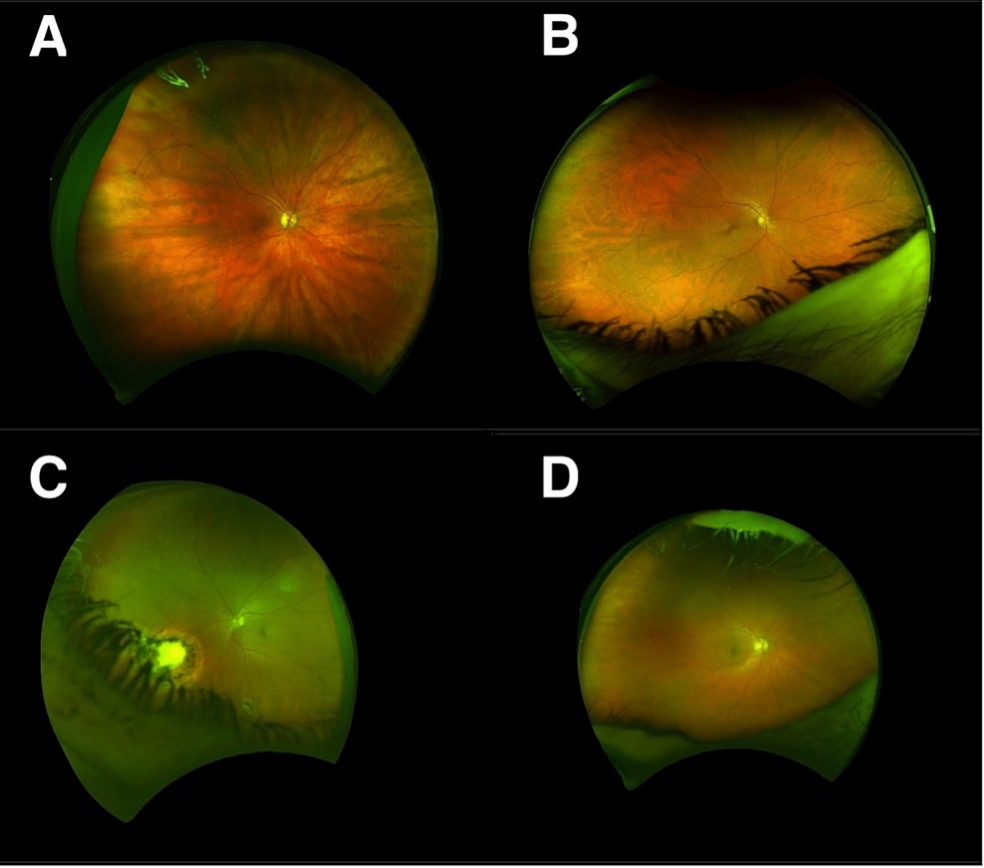
Images taken using Optos ultra-widefield imaging. (A) Right eye with obscuration score 0/12; no lesions. (B) Right eye with obscuration score 4/12; obscured by lids lashes and shadow; no lesions. (C) Left eye showing nasal scar and inferior angioma; obscuration score 2/12; obscured by lashes. (D) Left eye with obscuration score 2/12; obscured by lids.

The slit-lamp fundus examination showed 11/94 (11.7%) eyes positive for angioma compared to 12/94 (12.8%) following the Optos image analysis (Figure 3). There was a discrepancy in only one eye (1.1%) between the two groups. The Optos imaging picked up all the angiomas seen on the slit-lamp fundus examination, but in one image, an area of increased blood vessel tortuosity was identified, suggestive of a potential angioma outside the field of view of the image. Therefore, this eye was graded as positive for angioma, and the patient was brought back for a re-examination of the peripheral retina in the clinic. On repeat slit-lamp examination, no angioma was seen (false positive). Optos imaging identified all angiomas seen on the slit-lamp examination in this study. The null hypothesis is that the Optos imaging is inferior to the slit-lamp examination. Using a non-inferiority margin of 0.15 with a required power of 0.9, a sample size of 94 eyes in each arm was required [8]. As the Optos imaging identified all the angiomas seen on the slit lamp, the null hypothesis was disproved. Example images taken using the Optos camera are shown in Figure 4.

 

## Discussion

This audit assessed whether virtual retinal screening clinics in patients with VHL were as good as fundus slit-lamp examination in a cohort of patients not stratified for a priori risk. Our approach was different from a study by Smith et al., where they stratified VHL patients into a low-risk category and offered these patients ultra-widefield imaging as an alternative to slit-lamp screening [6]. By contrast, we included all patients who were available to come to the Eye Hospital for imaging after their VHL multidisciplinary clinic where they had had their fundus slit-lamp examination. In the audit by Smith et al., they included three groups: those with a family history of VHL disease but no active disease, those with an isolated tumour but with no gene mutation and those aged over 30 years without retinal angiomas. Of 80 patients attending the clinic, 44 met these criteria and were imaged with subsequent remote analysis. The images taken were deemed to be of sufficient quality by the expert operator. The authors suggest that retinal imaging, therefore, is a viable alternative to face-to-face examination. The authors did not have a control group, and as such, a sensitivity calculation was not made. As a consequence of their audit, patients deemed lower risk are now offered ultra-widefield imaging as an alternative to the slit-lamp examination. To the best of our knowledge, no other studies have investigated the viability of creating a remote virtual clinic for VHL patients by calculating the sensitivity of camera images. Our investigation included all patients presenting to the VHL multidisciplinary clinic rather than a lower risk cohort, and we offered both slit-lamp examination and remote imaging so that a definitive sensitivity calculation could be obtained between remote imaging and face-to-face examination. All of the eyes with angiomas within our cohort were identified by Optos ultra-widefield imaging. Example images taken using the Optos camera, along with interpretation, are shown in Figure 4. Results taken from these 94 eyes over 16 months suggest that Optos ultra-widefield imaging is as sensitive for identifying early retinal angiomas in VHL disease as the in-person, slit-lamp examination by a specialist ophthalmologist.

Optos imaging has other potential benefits over the slit-lamp examination. Optos imaging is faster and potentially easier in paediatric patients and creates a more detailed permanent record allowing for easier comparison between appointments and was preferred by patients in the Cambridge study [6]. Optos ultra-widefield images can image up to 80% of the retina, which is comparable to the slit-lamp examination unless indentation is carried out or a gonio fundus contact lens is used. Potentially, very peripheral angiomas that may not physically appear on imaging can be identified by the presence of suspicious feeder retinal blood vessels.

Retinal angioma management is to destroy the tumour, and as early tumour ablation is associated with significantly better outcomes, regular surveillance to identify tumours at the earliest possibility is key to this strategy [9]. Non-attendance rates at the Oxford VHL clinic have been recorded to be as high as 30%. Missed clinics can be difficult to reschedule imminently due to the infrequent nature of these specialist VHL screening clinics. This can result in significant delays before assessment, and therefore, tumours may show progression. Virtual clinics could address scheduling issues for patients who cannot attend the fixed monthly VHL multidisciplinary clinic. Virtual clinics have been successfully introduced into other subspecialty areas of ophthalmology, with high patient satisfaction rates [10]. Reduction of face-to-face contact at the slit lamp reduces risks to both clinicians and patients infected by airborne pathogens. During the COVID-19 pandemic, many face-to-face appointments were postponed or cancelled. By offering VHL patients virtual clinic assessments, surveillance can be continued and the risk of missing early angiomas is reduced.

The main limitation of concern was that of image obscuration. Optos images frequently had some degree of obscuration from lids, lashes or artefacts (Figure 2), and only 14% had zero obscuration. The average image taken shows an obscuration score of 2.4 out of a total of 12 retinal zones (Figures 1-2). We attempted to reduce the amount of obscuration by taping eyelashes to maintain a clear field of view. This was found to be acceptable by the patients. The possibility of using a speculum was also discussed; however, due to the small risk of causing a corneal abrasion and the need for topical anaesthesia, this was not adopted. Overall, obscuration in our Optos images did not reduce the sensitivity of Optos imaging in our cohort. This may be due to the ability of the retinal screening ophthalmologist to identify suspicious vessels even if the angioma itself is obscured (as was the case in two eyes, with one eye being false-positive). In our one false-positive finding on Optos photography, potentially suspicious vessels were identified, and it was thought that an angioma may be present, but the far peripheral view where the angioma might have been was not visible on the imaging. On repeat face-to-face examinations, no angioma was found. This issue was noted part-way through data collection, and as such, we did not change our protocol. In hindsight, the risk of creating trauma from speculum usage is minimal as highlighted by routine speculum usage in the paediatric clinical examination. Inoue et al. found that the use of a disposable speculum reduced obscuration from eyelashes in Optos imaging without causing pain or discomfort to the patient. They suggest this as a mechanism to increase the sensitivity of retinal lesion detection [11]. As such, we suggest the use of topical anaesthesia and a speculum to address the image obscuration issue that we faced.

## Conclusions

To conclude, we have shown that Optos imaging is non-inferior to the slit-lamp examination in the detection of retinal angiomas in this sample of 94 eyes. This study supports the provision of remote virtual VHL screening clinics using ultra-widefield imaging as a viable alternative to conventional face-to-face examination. Although in this study image obscuration did not impact sensitivity, we note that several images had a significant degree of image obscuration. An improvement in our protocol would be the usage of topical anaesthesia and eyelid retractors to reduce image obscuration on Optos imaging.
